# Large-scale screening of clinical assessments to distinguish between states in the Integrated HD Progression Model (IHDPM)

**DOI:** 10.3389/fnagi.2024.1320755

**Published:** 2024-02-13

**Authors:** Zhaonan Sun, Jennifer Ware, Sanjoy Dey, Elif Eyigoz, Swati Sathe, Cristina Sampaio, Jianying Hu

**Affiliations:** ^1^IBM Research, Yorktown Heights, NY, United States; ^2^CHDI Management, Inc., Princeton, NJ, United States

**Keywords:** Huntington’s disease, clinical assessments, trial design, disease states, IHDPM

## Abstract

**Background:**

Understanding the sensitivity and utility of clinical assessments across different HD stages is important for study/trial endpoint selection and clinical assessment development. The Integrated HD Progression Model (IHDPM) characterizes the complex symptom progression of HD and separates the disease into nine ordered disease states.

**Objective:**

To generate a temporal map of discriminatory clinical measures across the IHDPM states.

**Methods:**

We applied the IHDPM to all HD individuals in an integrated longitudinal HD dataset derived from four observational studies, obtaining disease state assignment for each study visit. Using large-scale screening, we estimated Cohen’s effect sizes to rank the discriminative power of 2,472 clinical measures for separating observations in disease state pairs. Individual trajectories through IHDPM states were examined. Discriminative analyses were limited to individuals with observations in both states of the pairs compared (*N* = 3,790).

**Results:**

Discriminative clinical measures were heterogeneous across the HD life course. UHDRS items were frequently identified as the best state pair discriminators, with UHDRS Motor items – most notably TMS – showing the highest discriminatory power between the early-disease states and early post-transition period states. UHDRS functional items emerged as strong discriminators from the transition period and on. Cognitive assessments showed good discriminative power between all state pairs examined, excepting state 1 vs. 2. Several non-UHDRS assessments were also flagged as excellent state discriminators for specific disease phases (e.g., SF-12). For certain state pairs, single assessment items other than total/summary scores were highlighted as having excellent discriminative power.

**Conclusion:**

By providing ranked quantitative scores indicating discriminatory ability of thousands of clinical measures between specific pairs of IHDPM states, our results will aid clinical trial designers select the most effective outcome measures tailored to their study cohort. Our observations may also assist in the development of end points targeting specific phases in the disease life course, through providing specific conceptual foci.

## 1 Introduction

Disease-modifying therapeutic development for Huntington’s disease (HD) depends on the availability of appropriate outcome measures or clinical endpoints for use in clinical trials. Given the evolution of HD symptomatology over decades (Roos, 2010; Ross et al., 2014), the utility of specific outcome measures to discriminate between stages over the disease life-course will vary as a function of the disease stage under study. A temporal map of relevant clinical assessments at the different stages of HD will aid future clinical research and inform trial design.

To date, the endpoints in almost all HD clinical trials have been the various components of the Unified Huntington’s Disease Rating Scale (UHDRS^®^′99) (Kieburtz et al., 1996), a clinical scale that consists of multiple assessments from four domains: motor function, cognitive function, behavior, and functional capacity. Commonly used clinical assessment items from the UHDRS^®^′99 include the Total Motor score (TMS), Total Functional Capacity (TFC) score, and the Symbol Digit Modalities Test (SDMT). The composite UHDRS (cUHDRS) score (Schobel et al., 2017) has also been derived from the UHDRS^®^′99 as a potential endpoint to assess disease progression.

We recently reported the development of an Integrated Huntington’s Disease Progression Model (IHDPM) that comprehensively characterizes HD symptom progression (Ghosh et al., 2017; Sun et al., 2019; Mohan et al., 2022). Based on this model, HD clinical phenotypes can be segregated into nine clusters of increasing severity, defined by varying combinations of motoric, cognitive, and functional measurements. Each of the nine clusters can be considered as a disease ‘state’ within the clinical phenotype. This differs from a staging system, which is typically built on disease prognostic milestones that define consecutive periods distinguished qualitatively by occurrence of new events. Using the IHDPM, we aim to systematically evaluate the ability of each of the more than 2,000 individual clinical assessment items that were recorded in the integrated dataset (used to generate the IHDPM) to characterize and distinguish between different IHDPM states and, by extension, generate a temporal map of the clinical measures most likely to change between states spanning the HD life-course.

## 2 Methods

### 2.1 Study dataset

The creation of the integrated dataset and development of the IHDPM has been previously described in detail (Ghosh et al., 2017; Sun et al., 2019; Mohan et al., 2022). Briefly, data from four observational studies {PREDICT-HD [Neurobiological Predictors of Huntington's Disease (Predict-Hd) (NCT00051324), n.d.], REGISTRY [Registry – an Observational Study of the European Huntington's Disease Network (Ehdn) (NCT01590589), n.d.], TRACK-HD & ON (Tabrizi et al., 2011, 2013) and Enroll-HD [Enroll-Hd: A Prospective Registry Study in a Global Huntington's Disease Cohort (NCT01574053), n.d.]} were curated into an integrated database, including clinical assessments across four symptom domains (motor, function, cognition, behavior), recorded longitudinally. Records of participants who enrolled in multiple studies were linked by their ID code (HDID). These studies include both people with HD (PwHD) and controls, but only PwHD were included in the current analysis. The starting sample for analysis comprised 64,758 observations from a sample of *N* = 18,941 individuals (Table 1). Discriminative analyses were based on a subsample limited to individuals observed to transition between states (i.e., observations in at least two states), resulting in an analysis set of *N* = 3,790 unique participants (Table 1).

**Table 1:** Participant characteristics.

Characteristic	Descriptive statistics (full)	Descriptive statistics (analysis)
Total sample (N)	18,941	3,790
Sex; N (%)^1^		
Female	10,114 (53.4%)	1,940 (51.2%)
Male	8,554 (45.2%)	1,825 (48.2%)
Age (baseline); mean, SD	47.8 (13.8)	48.9 (12.5)
Region; N (%)		
Europe	14,249 (75.2%)	3,330 (87.9%)
North America	4,009 (21.2%)	406 (10.7%)
Australasia and Asia	546 (2.9%)	47 (1.2%)
Latin America	137 (0.7%)	7 (0.2%)
Ethnicity; N (%)		
White	16,707 (88.2%)	3,470 (91.6%)
Others	2,234 (11.8%)	320 (8.4%)
CAG length; mean, SD	43.9 (4.3)	44.5 (4.6)
CAP score (baseline)	100.6 (24.8)	106.7 (17.0)
Number of visits/observations; mean, SD	3.4 (2.5)	6.0 (2.3)
Observation period (years); mean, SD	2.9 (2.9)	5.9 (2.6)

1 Sex data missing for *N* = 273 in full sample and *N* = 25 in analysis sample.

### 2.2 Candidate clinical measures

The Integrated HD dataset contained variables including *‘static’* variables (values do not change over time, e.g., certain demographic variables and genetic information including CAG repeat length), *‘interval’* variables (which have a defined start and end time, e.g., existence of comorbidities and use of medications) and *‘dynamic’* variables (values may change at different study visits, e.g., clinical assessments). This analysis focused on the 2,472 dynamic variables – comprising individual and summary items from multiple clinical rating scales – (Supplementary Table S2) which make up the majority of variables in the Integrated HD dataset. Although the machine-learning derived IHDPM did not include behavioral measures (Mohan et al., 2022), in this analysis we also include and assess variables from the Problem Behaviors Assessment (long), as well as the UHDRS Problem Behaviors Assessment (short; PBA-s) which produces summary scores for depression, irritability/aggression, psychosis, apathy and executive function.

### 2.3 IHDPM system and state assignment

**Figure 1:**
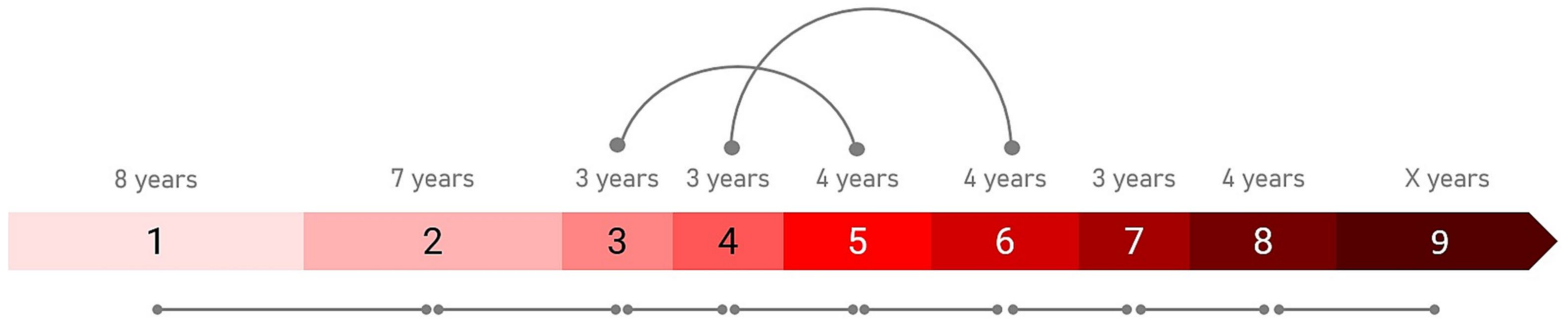
Integrated HD Progression Model (IHDPM) state comparisons. The pairwise comparisons examined between states are indicated. All sequential pairs were examined (e.g., S1 vs. S2). State skipping was observed in individual participant trajectories during the transition period (i.e., states 3 to 5), thus non-sequential pairwise comparisons were also examined in this phase (i.e., S3 vs. S5 and S4 vs. S6). The expected state duration time is also indicated.

**Figure 2:**
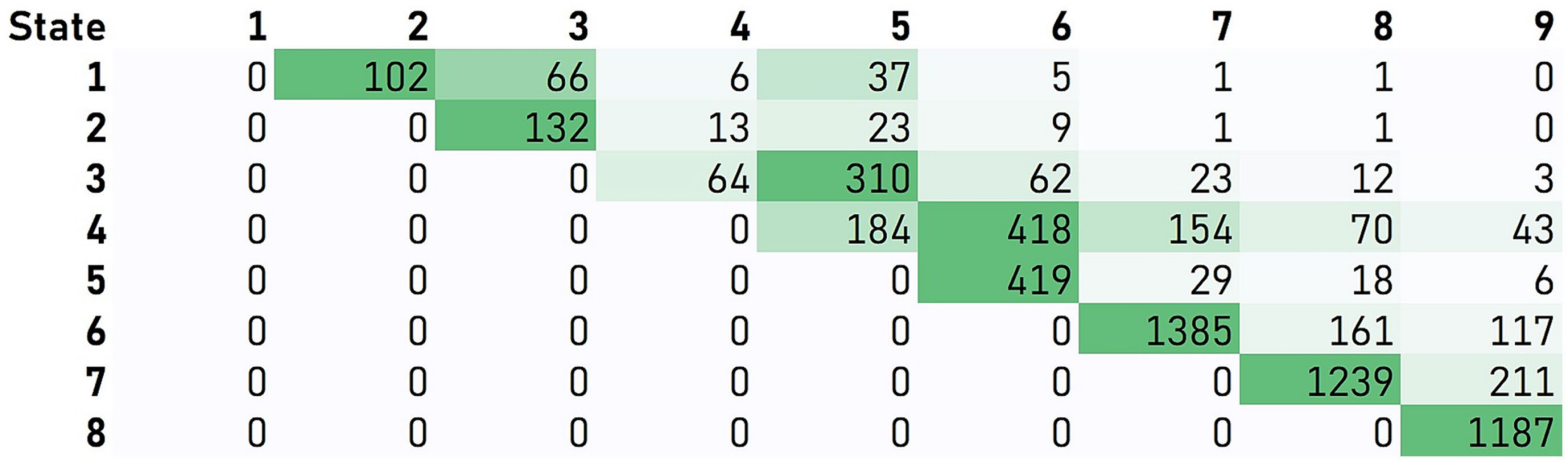
Participant transitions between IHDPM states. Transition pathways out of individual states were examined in those participants with sufficient longitudinal data (i.e., observations in at least two states). Matrix values and associated color-formatting (green scale) represent the frequency of individuals moving directly from the state indicated on the vertical to the state indicated on the horizontal. For most states (i.e., 1, 2, and 5–8), participants predominantly transitioned directly from one state to the immediate sequential state. State skipping was commonly observed from states 3 and 4.

The IHDPM selected 44 clinical measures during the model development (Mohan et al., 2022) and, separated HD progression into nine distinct states, spanning ∼36 years (encompassing clinical motor diagnosis) (Figure 1). In case of longitudinal observations, the IHDPM model maps each visit to the most likely disease state (from one to nine), based on data from all observations up to that visit. We applied the IHDPM model to all PwHD in the Integrated HD dataset and obtained disease state assignment for all study visits. Recognizing that individuals do not necessarily transition through IHDPM states sequentially – particularly across the ‘transition’ states (i.e., 3, 4, 5) during which clinical motor diagnosis is expected to occur – we examined transition pathways out of individual states in those participants with sufficient longitudinal data (i.e., observations in at least two states) (Figure 2). This analysis informed the state pairwise comparisons examined.

### 2.4 Statistical analysis

Pairwise comparisons were conducted between clinical measure scores observed in specific pairs of IHDPM states. In order to evaluate the discriminative power of each of the clinical measures in distinguishing the IHDPM states, we used Cohen’s effect size (Cohen’s D). For a pair of disease states i and j, the effect size of a clinical assessment f is calculated as

$$Df,\left(i,i+1\right)=x¯i+1-x¯i/\mathrm{Spool}$$

where x_¯i + 1_ − x_¯i_ are the sample means of assessment *f for all available observations* in state *i* and *j* respectively, and *S_pool_* is the pooled standard deviation of the two groups, and is calculated as

$$S_{\mathrm{pool}}=\sqrt{\frac{\left(n_{i}-1\right)s_{i}^{2}+\left(n_{j}-1\right)s_{j}^{2}}{n_{i}+n_{j}}}$$

where ni and s2 denote the sample size and the sample variance of assessment f for observations assigned to state *i.*

For each pair of states, the Cohen’s D effect size of clinical assessment items were ranked by the absolute values of their effect sizes. In this study, an assessment item f is called to be a discriminative clinical measure between states *i* and j if the corresponding Cohen’s D is greater than 0.8 (large effect size). Among the discriminative assessments with large effect sizes, those that were unique to a specific pair of states were named the signature assessments of the two states. Not all variables were consistently available in all four studies and missing values were common in these observational studies. For each clinical variable *f*, the Cohen’s D was calculated for pairs of states *i* and *j* only if there was a sample size of ≥30 in both states *i* and *j.*

In addition to examination of all consecutive state pairs (e.g., state 1 vs. 2), we also examined non-consecutive state pairs reflective of the most frequently observed trajectories from each state (e.g., state 3 vs. 5), informed by our analysis of participant transitions between IHDPM states (see Figures 1, 2). For most states (i.e., 1, 2, and 5–8), participants predominantly transitioned directly from one state to the immediate sequential state. However, state skipping was observed. The most frequently observed transition from state 3 was to state 5, and from state 4 to state 6. As such, we performed pairwise comparisons of all assessment items between states 3 vs. 5, and 4 vs. 6. We note that other, less common, transitions were observed, for example, state 1 to state 3, and state 4 to state 7 (Figure 2); these state pairs were not analyzed.

Our analysis sample for each IHDPM state pair was limited to individuals with observations in each of the states of interest, where the individual transition trajectory was from state A to state B (i.e., the two states indicated in the pairwise comparison). This limited our discriminative analysis sample to a total of 3,790 unique participants.

To aid comparisons with other studies, we also estimated annualized effect sizes that take into account the differing lengths of time people with HD spend in each state (ranging from 3 to 8 years; see Figure 1; Mohan et al., 2022). Annualized effect sizes were estimated as *Effect size of assessment between state i and state j / expected duration of state i (in years).*

## 3 Results

The Integrated HD dataset provides a diversified sample of 18,941 PwHD for whom IHDPM states have been calculated. Our discriminative analysis set was limited to a subsample of those 3,790 individuals who were observed to transition between states. Table 1 summarizes the key demographic and genetic characteristics for the original and analysis samples, as well as visit counts and observation periods.

Supplementary Table S1 summarizes the IHDPM state pairwise comparison results, ordered by their absolute effect size, limited to those assessment items with an absolute effect size of ≥0.8. Signature assessments – unique to a specific pair of states – are highlighted. A complete set of results, including results for all clinical measures by state pair, are provided in Supplementary Table S2.

Between the early-disease states (states 1 vs. 2, 2 vs. 3, 3 vs. 4), UHDRS motor assessment measures predominantly had the highest discriminative power, with effect sizes for TMS, diagnostic confidence level (DCL), and individual chorea items ranking highest. One additional and notable source of discriminatory items for the state 1 vs. 2 comparison was the SF-12 assessment; almost all individual dimension scores (e.g., mental health, general health, social functioning) featured as discriminatory items of large effect size, and many of these were unique to this state pair. Several SF-12 dimension scores were also identified for the state 3 vs. 4 comparison. For the cognitive domain, a completion indicator variable for the UHDRS cognitive verbal fluency test (letters) was identified for the state 1 vs. 2 comparison. Stroop Color and Stroop Word Reading tests (total correct) were identified for state 2 vs. 3, alongside independence score and UHDRS Q80 – a holistic diagnostic indicator considering all disease domains. Stroop Color, Stroop Word, and the Symbol Digit Modality Test (all UHDRS cognitive components) were identified for state 3 vs. 4, alongside UHDRS Q80. The Global Clinical Impression score also featured as an item of large effect size, not only for these first three consecutive state pairs but for every state pair examined.

In the IHDPM staging system, states 3 to 5 belong to the so-called transition phase, during which most individuals experience clinical motor diagnosis (DCL = 4) (Sun et al., 2019; Mohan et al., 2022). In light of the transition pathways most commonly observed in this phase – with individuals typically transitioning directly from states 3 to 5, and from 4 to 6 – we focus on these non-sequential pairwise comparisons here. The best discriminators between states 3 and 5 were UHDRS functional measures (e.g., TFC score, independence score, and functional assessment score) alongside UHDRS motor items (e.g., TMS and DCL). Certain individual UHDRS TFC and Function items also ranked highly, including occupation, employment, domestic chores, and finances. Two items from the Caregivers Quality of Life (CQoL) assessment also feature – frustration by misconceptions of HD relatives, and discrimination toward them; these are signature assessment items unique to this state pair. A very long list of discriminators with large effect sizes were observed between states 4 and 6; functional (TFC score) and motor (TMS) measures ranked highest, but cognitive measures were also highlighted (Stroop Color, Stroop Word, SDMT), and many ‘signature’ measures were also observed, including laundry and automobile operation items in the UHDRS functional domain. Two PBA (long) measures – quality of work (severity; frequency) – also feature uniquely to this state pair. Between states 4 and 5 – a less common but still frequently observed state transition – the best discriminators are almost entirely functional. Total and individual item scores from the UHDRS TFC and Function/Independence assessments heavily feature, alongside three items from the WPAI-SHP – relating to employment and the impact of HD on regular activities – which are unique to this state pair. Note that DCL is *not* flagged as a discriminator of large effect for this pairwise contrast, or any subsequent pairs.

State 6 marks the beginning of the late-disease phase of the IHDPM staging system (Mohan et al., 2022). From state 5 to state 6, the list of discriminative assessments includes measures principally from the motor domain, with effect sizes in UHDRS TMS and total bradykinesia score ranking highest. From state 6 to state 9, the lists of discriminative measures include those from motor, cognitive, and functional domains, with an increasing emphasis on functional items discriminating between the later states. We identified the supervision of children without help item (UHDRS Function) and self-care (frequency) from the PBA (long) as signature items for state 6 vs. 7, and signature measures discriminating state 8 from state 9 included care level (functional; UHDRS TFC), completion of certain assessments (Trailmaking test, Timed Up and Go), receipt of crutches/sticks in the last 6 months (CSRI), MMSE total score (MMSE), ocular pursuit scores; vertical and horizontal (UHDRS Motor), and the ‘construction’ score from the Dementia Rating Scale 2.

A large proportion of measures identified as state pair discriminators were UHDRS items. Table 2 presents the IHDPM state pairwise comparisons for core UHDRS variables across all state pairs examined.

**Table 2:** IHDPM state pairwise comparison results for key UHDRS items.

Item	Variable (Enroll-HD)	State pair									
		1 vs. 2	2 vs. 3	3 vs. 4	3 vs. 5	4 vs. 5	4 vs. 6	5 vs. 6	6 vs. 7	7 vs. 8	8 vs. 9
Motor score	motscore	**1.53**	**1.24**	**1.71**	**1.27**	0.49	**1.92**	**1.62**	**1.45**	**1.04**	**1.51**
Diagnostic confidence level	diagconf	**1.60**	**1.19**	**1.12**	**0.82**	0.47	0.61	0.45	0.24	0.15	0.21
Total functional capacity score	tfcscore	0.45	0.75	0.46	**1.97**	**2.33**	**2.06**	0.67	**1.25**	**1.60**	1.71
Functional assessment score	fascore	0.51	0.47	0.52	**1.61**	**1.72**	**1.48**	**0.82**	**1.26**	**1.42**	1.75
Independence scale	indepscl	0.59	**0.82**	0.34	**1.74**	**1.99**	**1.78**	0.73	**1.24**	**1.40**	1.58
Symbol digit modality test; total correct	sdmt1	0.65	0.73	**0.87**	0.52	0.25	**0.86**	**0.96**	**0.93**	**0.92**	0.91
Verbal fluency test (letters); total correct 3 min	verflt05	0.26	0.27	0.37	0.14	0.05	0.37	0.43	0.44	0.51	0.51
Stroop color naming; total correct	scnt1	0.59	**0.85**	**1.07**	0.72	0.12	**0.81**	**0.92**	**0.89**	**0.82**	0.99
Stroop word reading; total correct	swrt1	0.51	**0.90**	**0.92**	0.64	0.23	**0.83**	**0.95**	**0.90**	**0.88**	1.07
Stroop interference; total correct	sit1	0.58	0.68	**0.80**	0.42	0.02	0.69	0.75	**0.80**	0.72	0.72
PBA-s depression	depscore	NA	0.19	NA	0.03	0.26	0.16	0.04	0.06	NA	0.00
PBA-s irritability/aggression	irascore	NA	0.27	NA	0.15	0.14	0.09	0.03	0.06	NA	0.19
PBA-s psychosis	psyscore	NA	0.16	NA	0.19	0.06	0.07	0.05	0.10	NA	0.12
PBA-s apathy	aptscore	NA	0.20	NA	0.19	0.41	0.34	0.10	0.34	NA	0.33
PBA-s executive function	exfscore	NA	0.26	NA	0.10	0.33	0.30	0.06	0.18	NA	0.20

Effect sizes are presented only; complete results are presented in Supplementary Table S2. Large effect sizes (≥0.80) are indicated in bold typeface.

## 4 Discussion

We conducted large-scale screening of all available dynamic clinical measures in the Integrated HD dataset and evaluated their ability to distinguish between observations of individuals in different IHDPM states spanning the life course of the disease. As expected, based on the clinical course of HD, the clinical measures that best discriminate between the IHDPM states are somewhat heterogeneous across the HD progression pathway.

A large proportion of measures identified as the best state pair discriminators were UHDRS items. In the early-disease states (states 1 vs. 2, 2 vs. 3, 3 vs. 4) and early post-transition period states (5 vs. 6, 6 vs. 7), UHDRS motor assessments – most notably TMS – showed the highest discriminative power. UHDRS functional items did not become strong discriminators between states until the transition period, specifically emerging in the state 3 to 5 comparison, and demonstrating the greatest discriminatory power for distinguishing between the final states (7 vs. 8, 8 vs. 9). Cognitive assessments (specifically, Stroop Word Reading Test, Stroop Color Naming Test, and Symbol Digit Modality Test) showed good, although typically not as large, discriminative power between almost all state pairs examined, excepting state 1 vs. 2. No PBA-s items were identified as state discriminators of large effect, in line with previous findings that show little evidence of PBA-s measure variation across the disease life course (Marder et al., 2000; Tabrizi et al., 2013). Nevertheless, it is noted that apathy (and, to a lesser extent, executive function) have moderate discriminative power beginning in the transition period, also concordant with previous reports (Tabrizi et al., 2012).

In addition to the UHDRS assessments, several other assessments were also flagged as excellent state discriminators, with variation observed over the life course. For example, the Short Form Health Survey (SF-12; a self-reported general health and well-being questionnaire with several dimensions such as general health, social functioning, and mental health) was identified as a source of several items of excellent discriminatory power between the earliest disease states (state 1 vs. 2). The Caregivers Quality of Life (CareQoL) survey was identified as a source of items for discrimination between observations in states 3 vs. 5. Three items from the Workers Productivity and Activity Impairment (WPAI-SHP) questionnaire, relating to employment and the impact of HD on regular activities, were flagged for the state 4 vs. 5 comparison, all unique to this state pair. Several unique items from other assessments were also flagged as excellent discriminators between the last two states (8 vs. 9), including items from the Mini Mental State Examination (MMSE; a clinician-administered assessment of cognitive impairment), Dementia Rating Scale 2 (DRS-2; an assessment of overall cognitive function), and the Client Service Receipt Inventory (CSRI; a tool to measure socio-economic costs related to disease). Notably, one non-UHDRS item appeared consistently as a discriminator of large effect across every state pair examined – the Global Clinical Impression (GCI) score, perhaps reflecting the expertise and ability of trained investigators to capture subtle and otherwise difficult to quantify changes – emphasizing the importance of the gestalt evaluation.

For certain state pairs, single assessment items other than total or summary scores were highlighted as having excellent discriminative power. For example, for the transitionary state comparisons (i.e., states 3 vs. 5, 4 vs. 5, and 4 vs. 6), occupation, gainful employment (accustomed work; any), finances, and domestic chores – all singular items from the UHDRS TFC and Function assessments – were measures with high discriminative power. Employment – as assessed by the WPAI-SHP – also had high discriminative power in the state 4 vs. 5 comparison, as did employment status as assessed by the CSRI. This is in line with our previous findings (Mohan et al., 2022) and the PREDICT study (Beglinger et al., 2010), which suggested that work life is the first functional ability to be affected by disease progression. Such observations may assist in the development of end points targeting specific phases in the disease life course, through providing specific conceptual foci.

Our analyses have used the IHDPM states to indicate the different stages of the HD life-course. Although the IHDPM covers a relatively larger range of HD progression than traditional HD staging systems, its observational study basis does not cover the extremely early phase of the disease between birth and state 1, nor does it cover the period just prior to death. States 1 and 9 should not be regarded as the beginning and ending of the disease and the identified discriminative and signature clinical measures may not be appropriate for future research or trials targeting disease more than 20 years before or after clinical motor diagnosis. It is also important to highlight the expected durations of the nine IHDPM states are not equal (Sun et al., 2019; Mohan et al., 2022). Future researchers should take expected state durations into consideration when using the insights from this analysis to design new studies. Finally, we acknowledge that a new state-of-the art staging system has been developed since the IHDPM was published – specifically the HD Integrated Staging System (HD-ISS) (Tabrizi et al., 2022). Work is currently underway to map the nine IHDPM states to the four HD-ISS stages (i.e., 0, 1, 2, 3). The results of the current analyses, combined with this new mapping, will highlight clinical assessments/items that could help develop tailored assessments for these HD-ISS stages, which might later be adopted as trial endpoints.

We systematically evaluated the utility of thousands of clinical measures (including single items and summary scores) from dozens of clinical assessments in distinguishing between individuals in different IHDPM states spanning ~40 years of the HD life-course, encompassing early, transitory, and late stages of the disease. We demonstrate the importance and utility of the various UHDRS assessments in distinguishing between individuals in specific neighboring pairs of IHDPM states – including a reiteration of the utility of the UHDRS TFC (and other functional measures) as an excellent discriminatory measure – but only from the transitional states and beyond (i.e., states 3 to 5, and subsequent pairs). We also demonstrate the utility of *non*-UHDRS assessments in distinguishing between IHDPM states, such as the SF-12, which critically demonstrated excellent utility in distinguishing between the earliest phases of the disease examined. These results will aid future clinical research and may inform trial design.

## Data availability statement

Enroll-HD, TRACK, REGISTRY, and PREDICT-HD datasets are made available via the Enroll-HD platform: https://enroll-hd.org/for-researchers/access-data-biosamples/. Full results of the analyses presented in this paper are provided in the Supplemental tables.

## Ethics statement

The data used in this study were from the Enroll-HD, TRACK (TRACK-HD & Track-On HD), REGISTRY and PREDICT-HD studies. The protocols for all included studies were approved by the relevant ethical review committees for each site and all participants provided written informed consent for participation. The studies were conducted in accordance with the local legislation and institutional requirements.

## Author contributions

ZS: Conceptualization, Data curation, Formal analysis, Validation, Writing – original draft. JW: Supervision, Validation, Visualization, Writing – original draft. SD: Data curation, Formal analysis, Project administration, Writing – review & editing. EE: Data curation, Formal analysis, Project administration, Writing – review & editing. SS: Project administration, Supervision, Validation, Writing – review & editing. CS: Conceptualization, Formal analysis, Funding acquisition, Investigation, Methodology, Resources, Supervision, Visualization, Writing – review & editing. JH: Conceptualization, Formal analysis, Methodology, Resources, Supervision, Validation, Writing – review & editing.

## Supplementary material

SUPPLEMENTARY TABLE S1 IHDPM state pairwise comparison results. Assessment items with large absolute effect sizes (≥ 0.8) are listed in rank order. Signature assessments – unique to a specific pair of states – are highlighted in yellow. Results limited to items with a minimum of 30 observations in each state within the specified pair.

SUPPLEMENTARY TABLE S2 IHDPM state pairwise comparison results; all items. Assessment items (all) are listed in rank order by absolute effect size. Results limited to items with a minimum of 30 observations in each state within the specified pair.
